# NR3C1/GLMN-Mediated FKBP12.6 Ubiquitination Disrupts Calcium Homeostasis and Impairs Mitochondrial Quality Control in Stress-Induced Myocardial Damage

**DOI:** 10.3390/ijms26178245

**Published:** 2025-08-25

**Authors:** Jingze Cong, Lihui Liu, Rui Shi, Mengting He, Yuchuan An, Xiaowei Feng, Xiaoyu Yin, Yingmin Li, Bin Cong, Weibo Shi

**Affiliations:** Hebei Key Laboratory of Forensic Medicine, Collaborative Innovation Center of Forensic Medical Molecular Identification, Department of Forensic Medicine, Hebei Medical University, Shijiazhuang 050017, China; 23033100293@stu.hebmu.edu.cn (J.C.); 24033100367@stu.hebmu.edu.cn (L.L.); 22033100275@stu.hebmu.edu.cn (R.S.); 24033100353@stu.hebmu.edu.cn (M.H.); 24033100366@stu.hebmu.edu.cn (Y.A.); 24031100106@stu.hebmu.edu.cn (X.F.); yinxiaoyu05@163.com (X.Y.); 16000557@hebmu.edu.cn (Y.L.)

**Keywords:** stress, calcium ions, mitochondrion, myocardial injury

## Abstract

Excessive stress disrupts cardiac homeostasis via complex and multifactorial mechanisms, resulting in cardiac dysfunction, cardiovascular disease, or even sudden cardiac death, yet the underlying molecular mechanisms remain poorly understood. Accordingly, we aimed to elucidate how stress induces calcium dysregulation and contributes to cardiac dysfunction and injury through the nuclear receptor subfamily 3 group c member 1 (NR3C1)/Glomulin (GLMN)/FK506-binding protein 12.6 (FKBP12.6) signaling pathway. Using mouse models of acute and chronic restraint stress, we observed that stress-exposed mice exhibited reduced left ventricular ejection fraction, ventricular wall thickening, elevated serum and myocardial cTnI levels, along with pathological features of myocardial ischemia and hypoxia, through morphological, functional, and hormonal assessments. Using transmission electron microscopy and Western blotting, we found that stress disrupted mitochondrial quality control in cardiomyocytes, evidenced by progressive mitochondrial swelling, cristae rupture, decreased expression of fusion proteins (MFN1/OPA1) and biogenesis regulator PGC-1α, along with aberrant accumulation of fission protein (FIS1) and autophagy marker LC3. At the cellular level, ChIP-qPCR and siRNA knockdown confirmed that stress activates the glucocorticoid receptor NR3C1 to repress its downstream target GLMN, thereby preventing FKBP12.6 ubiquitination and degradation, resulting in calcium leakage and overload, which ultimately impairs mitochondrial quality control and damages cardiomyocytes. In conclusion, our findings reveal that stress induces myocardial damage through NR3C1/GLMN-mediated FKBP12.6 ubiquitination, disrupting calcium homeostasis and mitochondrial quality control, and lay a theoretical foundation for dissecting the intricate molecular network of stress-induced cardiomyopathy.

## 1. Introduction

The stress response serves as a crucial adaptive process for preserving physiological homeostasis. Nevertheless, prolonged or intense stress exposure can pathologically activate the stress response, disrupt cardiac functional stability through complex, multidimensional pathways, and ultimately lead to cardiac dysfunction, multiple cardiovascular conditions, or even cardiac-originated sudden death [1,2,3].

Calcium ion (Ca^2+^) dysregulation serves as a key factor in cardiac dysfunction, acting simultaneously as a central signaling messenger and a crucial effector molecule. Cardiomyocyte excitation–contraction coupling critically relies on tightly regulated cytoplasmic Ca^2+^ transients: extracellular Ca^2+^ influx initiates massive Ca^2+^ release from the sarcoplasmic reticulum via type 2 ryanodine receptors (RyR2), markedly elevating intracellular Ca^2+^ levels to trigger myofilament sliding and cardiac contraction. Thereafter, Sarco/Endoplasmic Reticulum Ca^2+^-Transporting ATPase 2 (SERCA2) actively transports Ca^2+^ back into the sarcoplasmic reticulum, while the Na^+^/Ca^2+^ exchanger mediates Ca^2+^ extrusion, facilitating cardiac relaxation and resetting the system for the next contraction cycle [4,5,6]. Research has indicated that abnormal intracellular Ca^2+^ levels not only compromise cardiac mechanical performance but also disturb mitochondrial dynamics (e.g., fusion–fission imbalance), initiating apoptosis via mitochondrial pathways and resulting in cardiomyocyte damage [7,8,9,10]. Thus, perturbations in Ca^2+^ balance can directly compromise myocardial contractility, diastolic function, and electrical stability, serving as a fundamental pathological mechanism for cardiac dysfunction and damage.

FK506-binding protein 12.6 (FKBP12.6) serves as an essential molecular partner of RyR2, functioning like a “molecular lid” that selectively binds and stabilizes the closed state of the RyR2 channel [11,12]. Such stabilization is crucial for preserving Ca^2+^ homeostasis within cardiomyocytes, sustaining proper Ca^2+^ transients, and tightly controlling the cardiac contraction–relaxation cycle. Evidence suggests that reduced FKBP12.6 expression or its dissociation from RyR2 impairs channel gating, causing sarcoplasmic reticulum Ca^2+^ leakage and intracellular calcium overload [13,14,15]. Ubiquitination is a critical post-translational modification that can regulate protein expression levels through key processes such as protein degradation, signal transduction, endocytosis and lysosomal degradation, and intracellular trafficking [16,17]. Chronic or severe stress markedly stimulates the hypothalamic–pituitary–adrenal (HPA) axis, resulting in persistently increased glucocorticoid concentrations [18]. Yet, it is still unclear whether glucocorticoids mediate FKBP12.6 expression by affecting its ubiquitination, subsequently disrupting intracellular Ca^2+^ balance and causing cardiomyocyte damage. Therefore, building on well-established acute and chronic stress mouse models, this study elucidates how glucocorticoids regulate FKBP12.6 expression through the Nuclear Receptor Subfamily 3 Group C Member 1 (NR3C1, also known as glucocorticoid receptor), causing calcium overload, disrupting mitochondrial dynamics, and damaging cardiomyocytes. The goal of the study is to lay a theoretical foundation for a better understanding of the intricate molecular mechanisms involved in stress-induced cardiac damage.

## 2. Results

### 2.1. Stress-Induced Cardiac Dysfunction and Myocardial Cell Damage

**Figure 1:**
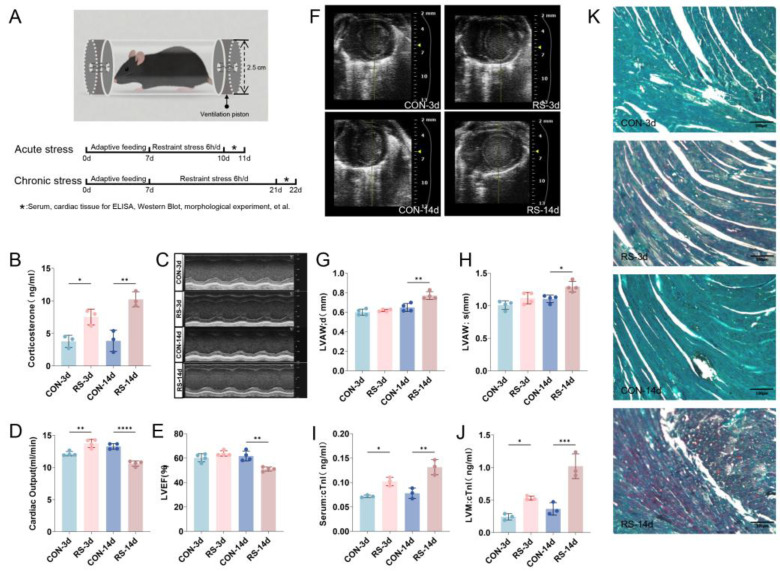
Stress triggers cardiac dysfunction and myocardial damage in mice. (**A**) Diagram of the RS model establishment. (**B**) Stress increases serum corticosterone level. Data are expressed as mean  ±  SEM, * *p* < 0.05, ** *p* < 0.01 vs. control, n  =  3. (**C**) M-mode ultrasound image of the mouse left ventricle. (**D**) Statistical analysis of cardiac output. Data are expressed as mean  ±  SEM, ** *p* < 0.01, **** *p* < 0.0001 compared to control, n  =  4. (**E**) LVEF. Data are expressed as mean  ±  SEM, ** *p* < 0.01, n  =  4. (**F**) B-mode ultrasound scan of the mouse left ventricle. (**G**,**H**) Thickness of the left ventricular wall in diastolic (LVAW; d) and systolic (LVAW; s) phases. Data are expressed as mean  ±  SEM, * *p* < 0.05, ** *p* < 0.01 vs. control, n  =  4. (**I**,**J**) Alterations in cTnI levels in mouse serum and LVM. Data are expressed as mean  ±  SEM, * *p* < 0.05, ** *p* < 0.01, *** *p* < 0.001 vs. control, n  =  4. (**K**) Chromotrope-2R brilliant green staining in the left ventricular myocardium. Red represents the damaged area, while green represents the normal tissue part. Scale bar = 100 μm.

Restraint stress (RS) is a classical model of psychological stress (Figure 1A). We first confirmed the successful establishment of the stress model by detecting serum corticosterone levels (Figure 1B). Then, we used echocardiography to evaluate the impact of stress on cardiac function. The results showed that cardiac output increased in acutely stressed mice, whereas left ventricular ejection fraction (LVEF) and cardiac output significantly decreased in chronically stressed mice. B-mode ultrasound revealed significant thickening of the left ventricular wall during both diastole and systole in chronically stressed mice. Meanwhile, M-mode echocardiography also showed increased cardiac output in acutely stressed mice and a significant reduction in LVEF and cardiac output in chronically stressed mice (Figure 1C–H). Biochemical assays revealed that stress elevated the levels of Cardiac Troponin I (cTnI) in both serum and left ventricular myocardium (LVM) tissue (Figure 1I,J). Moreover, chromotrope-2R brilliant green staining showed pathological changes such as ischemia and hypoxia in cardiomyocytes (Figure 1K). These results indicate that prolonged stress leads to cardiac dysfunction and myocardial injury.

### 2.2. Stress-Induced Mitochondrial Quality Control Disorder in Cardiomyocytes

**Figure 2:**
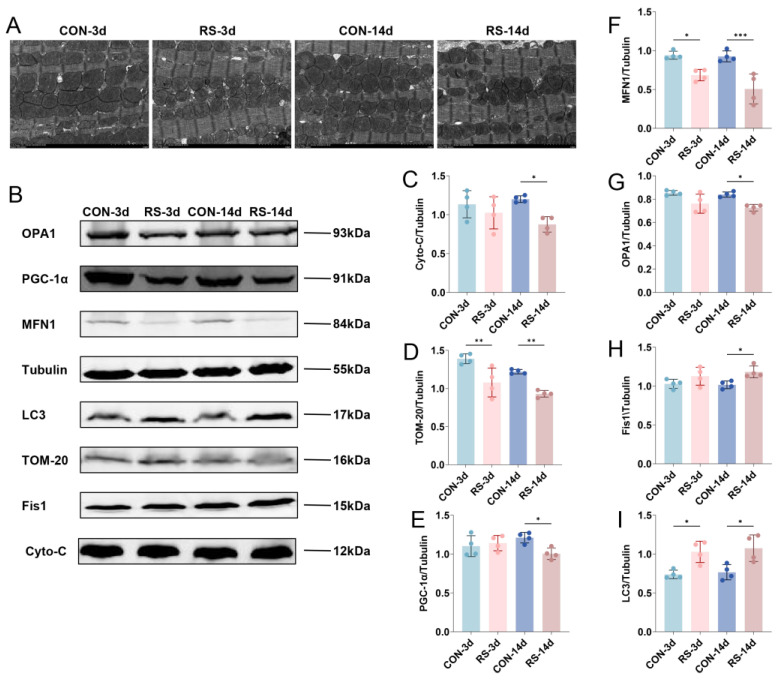
Stress causes mitochondrial dysregulation in the left ventricular myocardium. (**A**) Ultrastructural changes of mitochondria in cardiomyocytes. Scale bar = 2.0 μm. (**B**–**I**) Western blot and quantification of mitochondrial injury and quality control-related proteins in mouse myocardium. Data are presented as mean  ±  SEM, * *p* < 0.05, ** *p* < 0.01, *** *p* < 0.001 vs. control, n  =  4.

Mitochondria are sensitive organelles in cardiomyocytes for detecting stress-induced injury, and their structural and functional alterations are a major cause of cardiomyocyte damage [19]. Using transmission electron microscopy, we observed that stress induced mitochondrial swelling, cristae dissolution, and fragmentation in cardiomyocytes (Figure 2A). Meanwhile, stress also caused abnormal expression of cytochrome C (Cyto-c) and Translocase of Outer Mitochondrial Membrane 20 homolog (TOM-20) (Figure 2B–D), indicating that stress induced mitochondrial damage and dysfunction in cardiomyocytes. Mitochondrial morphology is primarily regulated by fusion and fission processes and tightly controlled by quality control mechanisms that determine mitochondrial function [20,21,22]. To further investigate the cause of mitochondrial damage, we examined key proteins involved in mitochondrial quality control and found that chronic stress reduced the expression of mitochondrial biogenesis regulator peroxisome proliferator-activated receptor gamma coactivator 1-alpha (PGC-1α) and fusion proteins mitofusin 1 (MFN1) and optic atrophy 1 (OPA1) in cardiomyocytes (Figure 2B,E–G), while the expression of mitochondrial fission protein-1 (FIS1) and autophagy marker microtubule–associated proteins light chain 3 (LC3) was increased (Figure 2B,H,I), indicating that stress reduces mitochondrial biogenesis and fusion but promotes fission and autophagy, leading to marked disruption of mitochondrial quality control in mouse cardiomyocytes.

### 2.3. GLMN-Mediated FKBP12.6 Ubiquitination Induced Intracellular Ca^2+^ Abnormalities

**Figure 3:**
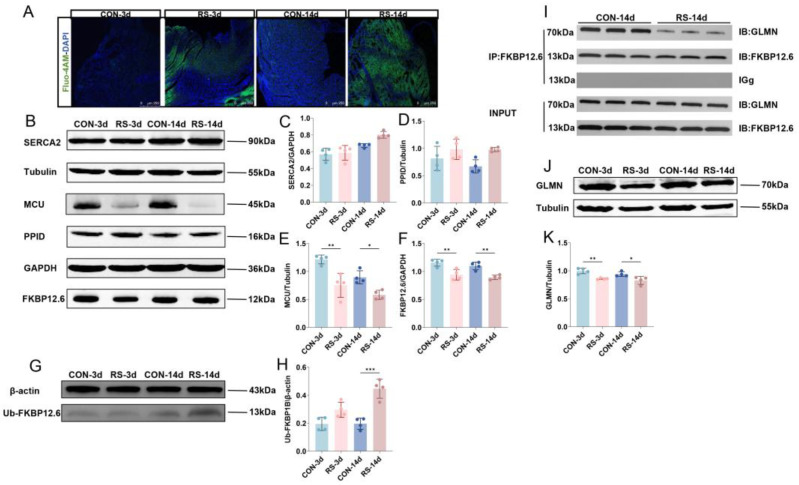
Aberrant regulation of GLMN-FKBP12.6 induces cytoplasmic calcium overload. (**A**) Co-staining of calcium fluorescent probe Fluo-4 AM and DAPI. Scale bar = 250 μm. (**B**–**F**) Western blot and quantification of calcium transport channel-related proteins in mitochondria (MCU, PPID) and endoplasmic reticulum (SERCA2, FKBP12.6). Data are presented as mean  ±  SEM, * *p* < 0.05, ** *p* < 0.01 vs. control, n  =  4. (**G**,**H**) Western blot and quantification of FKBP12.6 ubiquitination in mouse myocardial tissue. Data are presented as mean  ±  SEM, *** *p* < 0.001 vs. control, n  =  4. (**I**) Co-immunoprecipitation of FKBP12.6 and GLMN. (**J**,**K**) Western blot and quantification of GLMN in mouse myocardial tissue. Data are presented as mean  ±  SEM, * *p* < 0.05, ** *p* < 0.01 vs. control, n  =  4.

Abnormal cytosolic calcium levels are a key cause of impaired mitochondrial quality control [23,24,25]; we assessed calcium levels in cardiomyocytes and observed that both acute and chronic stress markedly elevated intracellular calcium levels (Figure 3A). Next, we analyzed critical calcium channel proteins in the sarcoplasmic reticulum (SR) and mitochondria (Figure 3B) and found no significant changes in SERCA2 or the mitochondrial permeability transition pore (mPTP)-regulating protein peptidyl-prolyl cis-trans isomerase D (PPID) after stress (Figure 3C,D), whereas expression of mitochondrial calcium uniporter (MCU) and FKBP12.6 proteins was markedly decreased (Figure 3E,F). FKBP12.6 acts as a regulatory protein for RyR2 channels in the SR, serving as a “molecular cap”. Loss of FKBP12.6 leads to RyR2 dysfunction, leaving the channel persistently open or leaky, causing elevated cytosolic calcium. To investigate the mechanism of FKBP12.6 downregulation, we observed that its ubiquitination was markedly increased following stress (Figure 3G,H). We subsequently searched for proteins that might regulate FKBP12.6 ubiquitination and found that GLMN, a regulator of the E3 ubiquitin ligase complex, bound to FKBP12.6 (Figure 3I), and GLMN expression was reduced in response to stress (Figure 3J,K), suggesting that both acute and chronic stress diminish GLMN-mediated protection against FKBP12.6 ubiquitination, resulting in its degradation in cardiomyocytes.

### 2.4. Stress Inhibits GLMN Transcription-Mediated Ubiquitination of FKBP12.6 Through NR3C1 Inhibition

**Figure 4:**
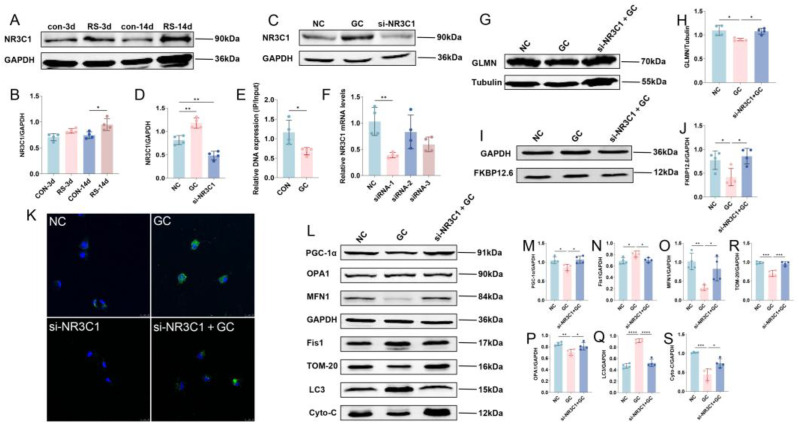
Stress inhibits GLMN transcription-mediated ubiquitination of FKBP12.6 through NR3C1 inhibition. (**A**,**B**) Western blot and quantification of NR3C1 in mouse myocardial tissue. Data are presented as mean  ±  SEM, * *p* < 0.05 vs. control. n = 4. (**C**,**D**) Western blot and quantification of NR3C1 in the cellular model. Data are presented as mean  ±  SEM, ** *p* < 0.01 vs. NC. n = 4. (**E**) CHIP-qPCR quantification of NR3C1 binding to GLMN. Data are presented as mean  ±  SEM, * *p* < 0.05 vs. control, n = 4. (**F**) Knockdown efficiency of si-NR3C1 in the cell model. Data are presented as mean  ±  SEM, ** *p* < 0.01 vs. NC, n = 4. (**G**,**H**) Western blot and quantification of GLMN in the cellular model. Data are presented as mean  ±  SEM, * *p* < 0.05, n = 4. (**I**,**J**) Western blot and quantification of FKBP12.6 in the cellular model. Data are presented as mean  ±  SEM, * *p* < 0.05 vs. NC. n = 4. (**K**) In the cellular model, calcium ions are labeled green using the probe Fluo-4 AM, while the cell nucleus is labeled blue using DAPI. Scale bar = 25 μm. (**L**–**S**) Western blot and quantification of mitochondrial damage and quality control-related proteins in the cellular model. Data are presented as mean  ±  SEM, * *p* < 0.05, ** *p* < 0.01, *** *p* < 0.001, **** *p* < 0.0001 vs. NC. n = 4.

Stress activates the HPA axis and markedly elevates circulating glucocorticoid levels (Figure 4A,B), prompting the investigation of whether this regulation occurs through glucocorticoid receptor-mediated transcriptional control of GLMN. Accordingly, we generated a cellular stress model by treating H9C2 cells with 500 μM glucocorticoid (GC) (Figure 4C,D) and used ChIP-qPCR to evaluate NR3C1 involvement in GLMN transcription. Glucocorticoid exposure significantly reduced GLMN mRNA expression in H9C2 cells (Figure 4E), suggesting that glucocorticoids repress GLMN transcription via NR3C1. We next established an H9C2 cell model with NR3C1 knockdown using siRNA (Figure 4F) and observed that reducing NR3C1 expression restored GLMN protein levels (Figure 4G,H) and significantly rescued stress-induced FKBP12.6 degradation mediated by ubiquitination (Figure 4I,J). In addition, we measured calcium levels in cardiomyocytes (Figure 4K) and assessed the expression of mitochondrial quality control-related proteins (Figure 4L), showing that silencing NR3C1 effectively blocked the stress-induced calcium overload, reversed the elevation of mitochondrial biogenesis protein PGC-1α (Figure 4M) and fission marker Fis1 (Figure 4N) caused by stress, and restored the stress-reduced expression of mitochondrial fusion proteins MFN1 and OPA1 (Figure 4O,P) and autophagy marker LC3 (Figure 4Q), thereby enhancing mitochondrial quantity (Figure 4R) and functional integrity (Figure 4S).

## 3. Discussion

Based on the successful establishment of acute and chronic RS models, we found that acute stress caused a compensatory increase in cardiac output, whereas chronic stress led to significantly reduced LVEF and ventricular wall thickening, accompanied by elevated levels of cTnI in serum and myocardium, along with pathological changes indicating myocardial ischemia and hypoxia, suggesting that chronic stress induces a critical transition from cardiac compensation to decompensation. Transmission electron microscopy analysis demonstrated distinct mitochondrial structural abnormalities, including swelling and cristae fragmentation. These ultrastructural alterations may compromise the spatial organization of electron transport chain protein complexes and impair mitochondrial Adenosine Triphosphate (ATP) synthesis efficiency. Concurrently, we observed significantly reduced expression levels of key mitochondrial regulators, including the biogenesis factor PGC-1α and fusion proteins MFN1 and OPA1. In contrast, the fission protein FIS1 and autophagy marker LC3 exhibited abnormal accumulation patterns. This widespread collapse of the mitochondrial quality control (MQC) network led to mitochondrial fragmentation, decreased quantity (TOM-20 reduced), and functional failure (Cyto-c release), directly compromising the energy supply capacity of cardiomyocytes, thereby providing an organelle-level pathological basis for cardiac decompensation [26,27].

Studies have shown that cytosolic calcium overload is a key driving force in triggering MQC disruption [28,29,30]. Our results showed a significant increase in intracellular calcium levels in cardiomyocytes after chronic stress, possibly due to pathological calcium leakage from sarcoplasmic reticulum RyR2 channels—ultimately caused by the loss of FKBP12.6 protein [11,12]. As a stabilizer of the RyR2 channel, FKBP12.6 deficiency leads to abnormal channel opening during diastole. Mechanistically, FKBP12.6 depletion results from its abnormally elevated ubiquitination, a process negatively regulated by the E3 ubiquitin ligase modulator GLMN. GLMN is a multifunctional protein whose key role includes acting as a negative regulator of specific E3 ubiquitin ligase complexes, effectively blocking the transfer of ubiquitin from E2-conjugating enzymes to substrates by shielding E2 binding sites, thereby preventing excessive ubiquitin-mediated substrate degradation [31,32]. Notably, RS significantly downregulated GLMN expression levels. Since GLMN physically interacts with FKBP12.6 and protects it from ubiquitination-mediated degradation, this stress-induced GLMN reduction consequently enhanced FKBP12.6 degradation through post-translational modifications. The subsequent depletion of FKBP12.6 resulted in RyR2 channel destabilization, triggering abnormal calcium leakage and ultimately disrupting intracellular calcium homeostasis. These findings suggest that disruption of calcium homeostasis initiates a vicious cycle of mitochondrial structural and functional damage [33,34]. Excess calcium enters mitochondria via the mitochondrial calcium uniporter, inducing aberrant opening of the mPTP, resulting in mitochondrial swelling, cristae disruption, and Cyto-c release [35].

We found that stress-activated HPA axis signaling led to a reduction in GLMN expression. Under chronic stress, persistent activation of NR3C1 significantly suppressed GLMN protein expression, resulting in loss of its protective function. Our validation experiments also confirmed that NR3C1 was strongly activated under stress conditions, which suppressed GLMN transcription by directly binding to its gene promoter region, thereby downregulating GLMN expression at both mRNA and protein levels. This series of events establishes the important role of the NR3C1/GLMN-FKBP12.6-RyR2 signaling axis in stress-induced myocardial injury.

Notably, sustained activation of the HPA axis during chronic stress and its mediation of elevated GC levels exert extensive effects on multiple organ systems [36,37,38]. The heart, as an organ with high energy demands and dependence on calcium homeostasis, relies on continuous, efficient mitochondrial energy supply and precise Ca^2+^ homeostasis to maintain its rhythmic contractile function. The NR3C1/GLMN-FKBP12.6-RyR2 signaling axis revealed in this study, along with the downstream disruption of calcium homeostasis and collapse of the MQC network, directly impacts the two fundamental pillars of cardiomyocyte survival and function: energy metabolism and electromechanical coupling [39,40].

Energy insufficiency resulting from mitochondrial functional failure directly impairs the driving force of myosin-actin cross-bridge cycling and SERCA2-mediated Ca^2+^ reuptake [41,42]. Coupled with pathological Ca^2+^ leakage through RyR2 channels, these defects manifest initially as reduced LVEF, decreased cardiac output, and ventricular wall thickening. If unmitigated, this progression culminates in heart failure over time [13], RyR2-mediated pathological Ca^2+^ leakage is a significant precipitant of delayed afterdepolarizations [43]. Cytosolic Ca^2+^ overload triggers abnormal action potentials, while the accumulation of reactive oxygen species and ATP deficiency from mitochondrial dysfunction further disrupts ion channel function, exacerbating myocardial electrical instability. This culminates in premature ventricular contractions, ventricular tachycardia, or even ventricular fibrillation [44]. Such stress-induced cardiac electrophysiological abnormalities represent a primary underlying cause of sudden cardiac death.

In summary, this study clarifies that NR3C1 directly interferes with cardiomyocyte calcium homeostasis through a non-classical pathway (GLMN-mediated ubiquitination regulation), subsequently triggering mitochondrial quality control disruption. This finding offers a novel mechanistic explanation for stress-induced myocardial injury and provides potential molecular markers for its diagnosis. Next, we will further investigate the specific E3 ligase complex and action sites involved in GLMN regulation of FKBP12.6 ubiquitination. Long-term intervention studies targeting this pathway will advance clinical translation, providing new strategies for precise prevention and treatment of stress-related cardiac diseases.

## 4. Materials and Methods

### 4.1. Stress Model Establishment

Male C57BL/6N mice (7–8 weeks, 22 ± 2 g) from Beijing Vital River Laboratory Animal Technology Co., Ltd. (Beijing, China) were acclimated for 7 days in SPF conditions (22 ± 2 °C, 12 h light/dark cycle). Animal protocols were approved by Hebei Medical University Ethics Committee (IACUC-Hebmu-2023011). Mice were randomly divided into four groups: control for 3 days (CON-3d), control for 14 days (CON-14d), restraint stress for 3 days (RS-3d), and restraint stress for 14 days (RS-14d), with n = 10 per group. Mice in the stress groups were restrained in cylindrical acrylic glass tubes (10 cm in length, 2.5 cm inner diameter) for RS. The tube body was provided with air holes and movable valves at both ends of the tube so that the mice could drill into the tube and could not move or turn freely [45]. RS groups were subjected to 6 h daily restraint (restraint tube model), where RS-3d represented acute stress with three consecutive days of restraint, and RS-14d represented chronic stress with fourteen consecutive days of restraint. Control groups experienced equivalent durations of food and water deprivation. At the endpoint, mice were anesthetized with 2% pentobarbital sodium (3.2 mL/kg, i.p.), blood was collected retro-orbitally, and they were euthanized. Hearts were excised and fixed in 4% paraformaldehyde, or rapidly frozen in liquid nitrogen and stored at −80 °C for future analysis.

H9C2 rat cardiomyocytes (STCC30008P) were purchased from Servicebio (Wuhan, China) and cultured in high-glucose DMEM (Viva Cell Biosciences, C3113-0500, Denzlingen, Germany) supplemented with 10% fetal bovine serum (ExCell Bio, FSP500, Shanghai, China) and 1% penicillin-streptomycin (Solarbio, P1400, Beijing, China). Cells were maintained in a humidified incubator at 37 °C with 5% CO_2_. For stimulation, 500 μM glucocorticoids were added to the complete medium for 24 h; all other conditions remained consistent with standard culture procedures.

Small interfering RNAs (siRNAs) targeting NR3C1, a negative control siRNA, and a fluorescently labeled siRNA were obtained from RiboBio (Guangzhou, China). Transfections were performed using riboFECT™ CP Transfection Reagent (RiboBio, R10035.7) according to the manufacturer’s protocol when the cell confluence reached 30–50%. After 48 h, transfection and knockdown efficiency were assessed by inverted fluorescence microscopy and RT-qPCR.

### 4.2. Echocardiographic Analysis of Cardiac Function

To ensure complete recovery of voluntary movement and minimize confounding variables, echocardiographic assessment was performed two hours post-RS modeling. Mice were lightly anesthetized with isoflurane and subjected to echocardiographic assessment of left ventricular systolic function using a Vevo 2100 system (VisualSonics, Toronto, ON, Canada) equipped with a 12–38 MHz linear transducer. Left ventricular systolic function was assessed via short-axis views obtained at the level of maximal left ventricular diameter to comprehensively evaluate RS-induced cardiac structural and functional alterations. B-mode imaging was used to evaluate ventricular wall thickness, while M-mode imaging assessed cardiac contractility. LVEF and cardiac output were quantitatively calculated by measuring left ventricle chamber dimensions during systole and diastole.

### 4.3. ELISA Detection of cTnI and Corticosterone

Mice were anesthetized via intraperitoneal injection of 2% pentobarbital sodium. Blood was collected retro-orbitally, and left ventricular myocardial tissues were harvested. Blood samples were centrifuged at 1000× *g* for 15 min at 4 °C. Serum and tissue samples were stored at −80 °C. According to the manufacturer’s protocols, serum and cardiac tissue homogenates’ cTnI levels, along with serum corticosterone levels, were quantified using a mice-specific cTnI ELISA kit (Cloud-Clone Corp, USEA478Mu, Wuhan, China) and a corticosterone ELISA kit (CAYMAN, 501320, Ann Arbor, MI, USA).

### 4.4. Chromotrope-2R Brilliant Green Staining

The isolated mouse heart was fixed in 10% formalin, and the tissue was subsequently dehydrated in steps of ethanol and embedded in paraffin. The heart wax block was sectioned into continuous sections (5 μm) for Chromotrope-2R brilliant green staining (LEAGENE, DK0007). Chromotrope-2R brilliant green staining is a sensitive method to assess cardiomyocyte injury. Slices were stained with Chromotrope-2R brilliant green staining for 10 min, fractionated three times in 2R fractionation solution for 5 min each, re-stained in bright green staining solution for 10 min, and finally observed under a light microscope (Olympus IX71; Mt. Olympus, Tokyo, Japan).

### 4.5. Changes in Mitochondria Morphology

Ventricular tissue was rapidly dissected from isolated mice hearts on ice, fixed in 2.5% glutaraldehyde (pH 7.4), and transferred to the Electron Microscopy Laboratory at Hebei Medical University. Samples were dehydrated through graded acetone series and embedded in epoxy resin. For ultrastructural analysis, at least 400 mitochondria were randomly selected from five distinct regions per sample.

### 4.6. Western BLOTTING

Mouse heart tissues were mechanically homogenized in a RIPA lysis buffer supplemented with protease and phosphatase inhibitors. After centrifugation at 14,000× *g* for 15 min at 4 °C, protein concentration was determined using a BCA assay kit. Proteins (8–12% SDS-PAGE) were separated and transferred onto PVDF membranes (Beyotime, FFP33/FFP24, Haimen, China) using a semi-dry transfer system (Trans-Blot Turbo). Membranes were blocked with 5% skim milk for 1 h, then incubated overnight at 4 °C with primary antibodies: OPA1 (1:1000, Proteintech, 27733-1-AP, Wuhan, China), PGC-1α (1:1000, Abclona, A12348), MFN1 (1:1000, Proteintech, 13798-1-AP), β-Tubulin (1:2000, Servicebio, GB11017-100), β-actin (1:1000, Servicebio, GB15003-100), GAPDH (1:5000, Servicebio, GB15004-100), LC3 (1:500, Proteintech, 14600-1-AP), Fis1 (1:1000, Proteintech, 10956-1-AP), TOM-20 (1:4000, Proteintech, 11802-1-AP), Cyt-C (1:1000, Abcam, ab133504), SERCA2 (1:1000, Proteintech, 27311-1-AP), MCU (1:1000, Proteintech, 26312-1-AP), PPID (1:500, Proteintech, 12716-1-AP), FKBP12.6 (1:2000, Proteintech, 15114-1-AP), GLMN (1:500, Proteintech, 32645-1-AP), and NR3C1 (1:1000, Proteintech, 24050-1-AP). Following incubation with IRDye 680RD anti-rabbit secondary antibody for 1 h at 37 °C, membranes were washed extensively with TBST. Protein bands were detected using the LI-COR Odyssey infrared imaging system, and relative expression levels were quantified using ImageJ software (v1.8.0, NIH, Bethesda, MD, USA).

### 4.7. Ca^2+^ Probe

Fluo-4, AM (Thermo Scientific, F14217, Waltham, MA, USA) was dissolved in 50 μL of DMSO (Thermo Scientific, D12345, Waltham, MA, USA) and diluted 1:1000 with HBSS (Report, RE0191, Shijiazhuang, China). Frozen sections or myocardial cell slides were incubated with the working solution at 37 °C in the dark for 30 min. Samples were then thoroughly washed with HBSS, counterstained with DAPI, and mounted using FluoroMount-G^®^. Images were acquired using a confocal laser scanning microscope (Leica SP8, Wetzlar, Germany).

### 4.8. Co-IP

Left ventricular myocardial tissue was lysed in an IP lysis buffer (Servicebio, G2038) supplemented with protease inhibitor (Servicebio, G2008). Protein A/G magnetic beads (Servicebio, G3657) were pre-washed with a binding buffer. For immunoprecipitation, 1.5 mg of protein lysate was incubated with 10 μL of anti-FKBP12.6 antibody (Santa Cruz Biotechnology, sc-136962, Dallas, TX, USA) and 30 μL of protein A/G beads overnight at 4 °C. After magnetic separation, the beads were washed with a binding buffer, then mixed with a 4× SDS loading buffer and boiled at 95 °C for 10 min. The supernatant was collected by magnetic separation and subjected to immunoblotting analysis.

### 4.9. ChIP

Cells were harvested and nuclei lysed to release chromatin, which was then fragmented by sonication. The sheared chromatin was incubated with a specific antibody against the target protein (Cell Signaling Technology, 3660S, Danvers, MA, USA), followed by addition of protein A/G magnetic beads (Cell Signaling Technology, 14209S, Danvers, MA, USA) to capture the antibody–protein–DNA complexes. After extensive washing to remove non-specific interactions, cross-links were reversed by heating at 65 °C in an SDS-containing buffer. Proteinase K digestion was performed to remove proteins, and the DNA was purified for subsequent qPCR analysis to identify target protein-bound genomic regions.

### 4.10. RNA Extraction and PCR

Total RNA was extracted from mouse heart tissue using TRIzol reagent (Invitrogen, Carlsbad, CA, USA), followed by chloroform extraction and isopropanol precipitation. As needed, complementary DNA (cDNA) was synthesized using the PrimeScript RT reagent kit (RR047A; Takara Bio, Shiga, Japan). Real-time quantitative PCR (qRT-PCR) or quantitative PCR (q-PCR) was performed using the SYBR Green PCR kit (RR820A; Takara Bio) to assess the mRNA expression of NR3C1 and GLMN (primers from Sangon Biotech, Shanghai, China). Gene expression levels were normalized to Gapdh and calculated using the 2^−ΔΔCt^ method. The primer sequences were as follows:

GAPDH: Forward primer: 5′-GACATGCCGCCTGGAGAAAC-3′;

reverse primer: 5′-AGCCCAGGATGCCCTTTAGT-3′.

GLMN: Forward primer: 5′-CACGAAACCCTTTGTGGCAG-3′;

reverse primer: 5′-GCAAAACACCGGAAAGGGTC-3′.

NR3C1: Forward primer: 5′-TCCCCCTGGTAGAGACGAAG-3′;

reverse primer: 5′-ACTGTAGCTCCTCCCCTCAG-3′.

### 4.11. Ubiquitination Assay

Cells were lysed using a RIPA lysis buffer (Report, RW0001, Shijiazhuang, China) supplemented with protease inhibitors (Report, RP-WA0120, Shijiazhuang, China). Ubiquitinated proteins were immunoprecipitated using an anti-ubiquitin antibody (1:100; Proteintech, 10201-2-AP) overnight at 4 °C, followed by incubation with protein A/G magnetic beads (Servicebio, G3657-1ML, Wuhan, China). After extensive washing, bound proteins were eluted by boiling in the sample buffer. The ubiquitination level of FKBP12.6 was assessed by Western blot using a specific anti-FKBP12.6 antibody (1:2000; Proteintech, 15114-1-AP).

### 4.12. Statistical Analysis

All data were analyzed using GraphPad Prism (version 9). Results are presented as mean ± SEM. Statistical significance was assessed using normality/lognormality tests followed by one-way ANOVA. *p* < 0.05 was considered statistically significant. All experiments were independently repeated at least four times.

## Data Availability

The datasets generated during and/or analyzed during the current study are available from the corresponding author on reasonable request.
